# Exosomes as Emerging Regulators of Immune Responses in Type 2 Diabetes Mellitus

**DOI:** 10.1155/2024/3759339

**Published:** 2024-02-29

**Authors:** Wei Zheng, Xin Ji, Qiao qiao Yin, Chensi Wu, Chengan Xu, Hongying Pan, Chun Wu

**Affiliations:** ^1^Center for General Practice Medicine, Department of Infectious Diseases, Zhejiang Provincial People's Hospital (Affiliated People's Hospital), Hangzhou Medical College, Hangzhou, Zhejiang, China; ^2^Department of Endocrinology, Chun'an First People's Hospital, Hangzhou, Zhejiang, China

## Abstract

Type 2 diabetes mellitus (T2DM) is a chronic metabolic disorder characterized by high blood glucose levels resulting from insulin resistance and impaired insulin secretion. Immune dysregulation-mediated chronic low-grade inflammation is a critical factor that poses a significant risk to the metabolic disorders of T2DM and its related complications. Exosomes, as small extracellular vesicles secreted by various cells, have emerged as essential regulators of intercellular communication and immune regulation. In this review, we summarize the current understanding of the role of exosomes derived from immune and nonimmune cells in modulating immune responses in T2DM by regulating immune cell functions and cytokine production. More importantly, we suggest potential strategies for the clinical applications of exosomes in T2DM management, including biomarkers for disease diagnosis and monitoring, exosome-based therapies for drug delivery vehicles, and targeted therapy for exosomes.

## 1. Introduction

Type 2 diabetes mellitus (T2DM) is a chronic metabolic disorder characterized by high blood glucose levels resulting from insulin resistance and impaired insulin secretion [1]. International Diabetes Federation reveals that the global prevalence of type 2 diabetes mellitus among adults was estimated to be 536.6 million (10.5%) in 2021, and this number will rise to 783.2 million (12.2%) by 2045 [2]. These findings highlight the significant burden of T2DM worldwide and emphasize the urgent need for effective prevention and management strategies to mitigate its impact on public health.

The pathogenesis of T2DM is complex and multifactorial, involving a combination of genetic, environmental, and lifestyle factors. In recent years, growing evidence suggests that immune dysregulation and chronic low-grade inflammation play a critical role in the development and progression of T2DM [3–5]. The immune system is a complex network of cells and molecules that protect the body against foreign invaders, such as viruses and bacteria. However, in T2DM, the immune system becomes dysregulated, leading to a chronic inflammatory state that contributing to insulin resistance and beta cell dysfunction [6]. This chronic inflammation also promotes the development of other diabetes-related complications, such as cardiovascular disease and neuropathy [7]. Therefore, understanding the role of immune responses in the pathogenesis of T2DM is crucial for developing new strategies for preventing and treating of this disease.

Exosomes are small extracellular vesicles secreted by various cells, including immune cells, and have emerged as essential regulators of intercellular communication and immune regulation [8]. Recently, there has been a growing interest in the potential role of exosomes as immune regulators in the context of T2DM. Exosomes contain a variety of bioactive molecules, including proteins, lipids, and nucleic acids, which can be transferred to recipient cells and modulate cellular functions. In particular, exosomes derived from immune cells have been shown to possess immunomodulatory properties and can suppress or enhance immune responses depending on their cellular origin and cargo [9]. Several studies have investigated the potential role of exosomes in T2DM pathogenesis and its associated complications. Exosomes derived from adipose tissue, pancreatic *β*-cells, and immune cells have been shown to contribute to insulin resistance, *β*-cell dysfunction, and chronic inflammation in T2DM [10, 11]. Conversely, exosomes derived from mesenchymal stem cells and regulatory T cells have anti-inflammatory and immunosuppressive effects and may have therapeutic potential for the treatment of T2DM [12, 13].

In summary, exosomes represent a promising area of immune research in T2DM and may provide new insights into the pathogenesis of the disease and potential therapeutic targets for its management. Further studies are needed to elucidate the role of exosomes in T2DM fully and to develop novel exosome-based therapies for this growing public health concern.

## 2. Definition and Characteristics of Exosomes

Exosomes range in size from 30 to 150 nm (nanometer) in diameter. They are formed by the inward budding of the plasma membrane, resulting in the formation of multivesicular bodies (MVBs) that eventually fuse with the plasma membrane and release their contents into the extracellular space [14]. Exosomes contain a variety of bioactive molecules, including proteins, lipids, and nucleic acids, which can be transferred to recipient cells and modulate cellular functions [15]. The cargo of exosomes varies depending on the cell of origin and can include signaling molecules, enzymes, transcription factors, and microRNAs, among others. The composition of exosomes is also dynamic and can be modulated by various stimuli, such as stress, inflammation, and disease [16].

## 3. Biogenesis and Release of Exosomes

**Figure 1:**
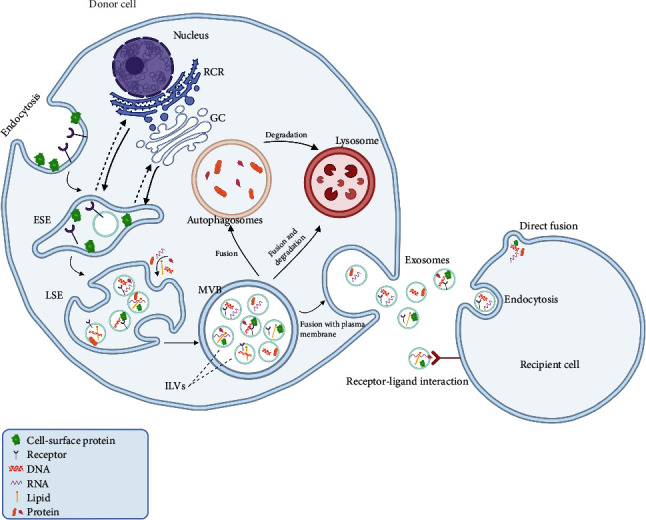
The biogenesis of exosomes and microvesicles. Microvesicles are formed through direct budding from the plasma membrane, while exosomes originate from the endosomal system. The biogenesis of exosomes begins with formation of early-sorting endosomes through endocytosis and plasma membrane invagination. During this process, extracellular materials can enter the endosomes along with cell-surface proteins. These early endosomes mature into late-sorting endosome, which generate intraluminal vesicles (ILVs) by budding inward from the endosomal membrane. These ILVs are the future exosomes. Late-sorting endosomes develop into multivesicular bodies (MVBs) with the accumulation of ILVs. MVBs can be transported to the plasma membrane and release exosomes by exocytosis. Alternatively, MVBs can fuse with autophagosomes or lysosomes and participate in the degradation pathway. The exosomal membrane maintains the same topological orientation as the plasma membrane because of the double invagination process. Exosomes can be taken up by recipient cells through various mechanisms, including endocytosis, receptor-ligand interaction, and direct fusion with the recipient cell membrane.

Exosomes are generated through a complex process of intracellular trafficking and fusion events involving the endosomal compartment and plasma membrane. The biogenesis of exosomes begins with forming early endosomes, which subsequently mature into late endosomes, also known as MVBs [17]. MVBs are characterized by the presence of intraluminal vesicles (ILVs) that are formed by the inward budding of the limiting membrane. ILVs contain diverse biomolecules, including proteins, lipids, and nucleic acids. The formation of ILVs is orchestrated by a group of proteins collectively known as the endosomal sorting complex required for transport (ESCRT) machinery [18]. The ESCRT machinery recognizes ubiquitinated proteins on the cytosolic face of the endosomal membrane. It recruits other ESCRT components to facilitate the budding of ILVs into the lumen of the MVBs. Once formed, the MVBs can either fuse with lysosomes for degradation or traffic to the plasma membrane for exosome release [19]. Below is a presentation of the process of biogenesis and the primary characteristics of exosomes (Figure 1).

Exosome release occurs through the fusion of the MVBs with the plasma membrane, leading to the extracellular release of exosomes. The regulation of exosome release is a complex process involving coordinating various signaling pathways, including Rab GTPases, SNARE proteins, and calcium ions. For example, Rab27a and Rab27b are both family members of Rab GTPases, which are found to function in multivesicular endosome (MVE) docking at the plasma membrane. The size of MVEs is strongly increased by Rab27a silencing, whereas MVEs were redistributed towards the perinuclear region upon Rab27b silencing. In addition, silencing two known Rab27 effectors, Slp4 (also known as SYTL4, synaptotagmin-like 4) and Slac2b (also known as EXPH5, exophilin 5), inhibited exosome secretion and phenocopied silencing of Rab27a and Rab27b, respectively [20]. SNARE proteins like syntaxin-4, SNAP-23, and VAMP-7 can promote exosome secretion, and their SNARE complex can drive membrane fusion in vitro [21]. Ca^2+^-channel protein ORAI1 can regulate the function of MLPH and SLP-a, two proteins related to controlling intracellular calcium ion concentration, thereby affecting the secretion of PD-L1 exosomes [22]. Once released into the extracellular space, exosomes can interact with recipient cells and deliver their cargo, thereby modulating cellular functions [23].

In summary, the biogenesis and release of exosomes involve a complex series of intracellular trafficking and fusion events that culminate in the extracellular release of small extracellular vesicles loaded with diverse biomolecules. The regulation of exosome biogenesis and release is a dynamic process that various stimuli, including stress, inflammation, and disease, can modulate.

## 4. Exosomes in Intercellular Communication

Exosomes have emerged as essential regulators of intercellular communication by transferring biomolecules, such as proteins, lipids, and nucleic acids, between cells. They can interact with recipient cells through various mechanisms, including receptor-ligand interactions, direct fusion with the plasma membrane, or endocytosis [24] (Figure 1). The cargo of exosomes can modulate cellular functions in recipient cells, including gene expression, protein synthesis, and cell signaling. Exosomes derived from immune cells have been shown to possess immunomodulatory properties and can suppress or enhance immune responses depending on their cellular origin and cargo [25, 26].

Exosomes have been shown to play essential roles in various physiological and pathological processes, including immune regulation, inflammation, tissue repair, and metabolism [27–29]. In the immune system, exosomes derived from immune cells can modulate the function of other immune cells and influence the outcome of immune responses. Inflammation is associated with changes in exosome cargo and can produce proinflammatory factors that contribute to the pathogenesis of various diseases. In tissue repair, exosomes can deliver growth factors that enhance tissue regeneration and repair. In metabolism, the cargo of exosomes can modulate cellular functions related to metabolism, such as glucose uptake, lipid metabolism, and insulin sensitivity. The cargo of exosomes is dynamic and can be modulated by various stimuli, suggesting that exosomes may represent a promising area of research for developing novel diagnostic and therapeutic strategies for various diseases.

## 5. Impact of Exosomes from Immune Cells on Immune Responses in T2DM

**Figure 2:**
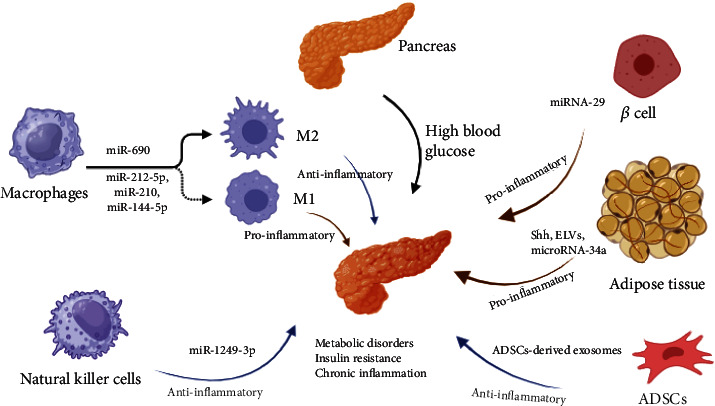
Mechanism of exosomes from immune and nonimmune cells on immune responses in T2DM. Shh: circulating sonic hedgehog; ELVs: exosome-like vesicles; ADSC: adipose-derived stem cell.

Exosomes play an essential role in modulating immune responses in T2DM by regulating immune cell function and cytokine production in several ways. Exosomes derived from immune cells can induce proinflammatory and anti-inflammatory responses depending on the context, such as proteins, lipids, and nucleic acids (Figure 2).

### 5.1. Macrophages

Macrophages undergoing polarization into either M1 or M2 subtypes exhibit proinflammatory or anti-inflammatory functions, respectively [30]. For example, obesity will trigger the polarization of macrophages in various organs, such as the pancreatic islets. Multiple studies have shown that during obesity-related dysfunction of beta cells, there is a general shift in macrophage polarity towards the M1-like phenotype [31, 32]. Exosomes secreted by inflammatory M1 macrophages impaired *β*-cell insulin secretion in an exosome-dependent manner. miR-212-5p was notably upregulated in M1-Exos and HFD-Exos, which impaired *β*-cell insulin secretion by targeting the sirtuin 2 gene and regulating the Akt/GSK-3*β*/*β*-catenin pathway in recipient *β*-cell [33]. The development of obese diabetes in mice is facilitated by miR-210 originating from adipose tissue macrophages, which regulate glucose uptake and mitochondrial complex IV activity by targeting the expression of the NADH dehydrogenase ubiquinone 1 alpha subcomplex 4 (NDUFA4) gene [34]. Additionally, researchers indicated that exosomal miR-144-5p derived from diabetic bone marrow-derived macrophages (BMDM) can be transferred to bone mesenchymal stem cells, leading to suppressed Smad1 expression and reduced bone repair and regeneration in vitro and in vivo [35]. Finally, it shows that M2-polarized BMDM exosomes contain miRNA and can improve metabolic parameters when administered to obese mice [36]. In short, exosomes from macrophages polarized towards either M1 or M2 subtypes can significantly affect the development and treatment of type 2 diabetes mellitus and related complications.

Above all, current studies of exosomes from immune cells on immune responses in T2DM can be seen in Table 1.

### 5.2. Natural Killer Cells

One study suggests that natural killer (NK) cells may regulate insulin sensitivity and inflammation in type 2 diabetes. Specifically, NK-derived exosomes from lean mice were attenuated obesity-induced insulin resistance and inflammation in diabetic mice while enhancing insulin sensitivity and reducing inflammation in adipocytes and hepatocytes. The exosomal miRNA, miR-1249-3p, was identified as a vital mediator of these effects, as it can be transferred from NK cells to target cells via exosomes and directly targets SKOR1 to regulate glucose homeostasis. These findings suggest that targeting NK-derived exosomal miR-1249-3p may be a potential therapeutic strategy for treating insulin resistance in type 2 diabetes [37].

## 6. Impact of Exosomes from Nonimmune Cells on Immune Responses in T2DM

Insulin resistance (IR) in patients with T2D is manifested by decreased glucose transport and metabolism in adipose tissue and skeletal muscle and by impaired suppression of glucose output in the liver [38, 39]. In diabetic patients, the resistance of adipose tissue, muscle, and liver to insulin is a crucial pathophysiological event in disease worsening [40–42]. Ectopic accumulation of lipids in the liver and skeletal muscle disturbs the insulin signaling pathways, leading to reduced glucose usage in muscle and decreased glycogen synthesis in the liver. One of the undesirable consequences of muscle and liver IR is increased hepatic de novo lipogenesis and hyperlipidemia. Exosomes derived from nonimmune cells can also modulate immune responses in T2DM (Table 2).

### 6.1. Pancreatic *β*-Cell

One study revealed a crosstalk pathway between *β*-cells and macrophages mediated by the miRNA-29-TNF-receptor-associated factor 3 (TRAF3) axis. Mechanistically, miR-29 promotes the recruitment and activation of monocytes and macrophages, leading to inflammation and impaired glucose homeostasis via miR-29 exosomes in a TRAF3-dependent manner. These findings demonstrate the ability of *β*-cells to modulate systemic inflammatory tone and glucose metabolism through miR-29 in response to nutrient overload [43].

### 6.2. Adipose Tissue

Adipose tissue is a significant source of circulating exosomal miRNAs that can modulate gene expression in distant tissues, thus functioning as a novel form of adipokine [44]. Exosome-like vesicles (ELVs) derived from adipocytes contain several proinflammatory adipokines, such as tumor necrosis factor-alpha (TNF-*α*), macrophage colony-stimulating factor (MCSF), interleukin-6 (IL-6), monocyte chemoattractant protein-1 (MCP-1), macrophage migration inhibitory factor (MIF), and retinol-binding protein 4 (RBP-4) [45].

Circulating sonic hedgehog- (Shh-) positive exosomes were increased in type 2 diabetes patients, and high glucose and insulin increased the secretion of Shh-positive adipocyte-derived exosomes (ADEs). The ADEs carrying Shh induced proinflammatory or M1 polarization of macrophages through the Ptch/PI3K signaling pathway, which contributed to insulin resistance in adipocytes by decreasing the expression of insulin-resistant substrate-1 (IRS-1) and hormone-sensitive lipase (HSL) [46]. Exosomes derived from the adipose tissue of ob/ob mice stimulated macrophage activation via the TLR4/TRIF pathway, with the presence of RBP4 in these exosomes playing a vital role in this process. The obesity-related exosomes then targeted the macrophages to the liver and adipose tissues, where they secreted TNF-*α* and IL-6, resulting in insulin resistance. Moreover, the researchers confirmed that exosome-mediated macrophage activation impaired insulin function in myocytes [47]. Adipocytes secrete exosomal miR-34a, which inhibits the polarization of M2 macrophages by targeting Krüppel-like factor 4 (Klf4), a protein that promotes M2 macrophage polarization and monocyte differentiation. The study also showed that the dysregulated miR-34a/Klf4 axis in visceral fat is strongly linked to insulin resistance in obese individuals [10]. To summarize, adipocyte-derived exosomes carrying proinflammatory adipokines can activate macrophages, producing proinflammatory cytokines and insulin resistance.

### 6.3. Mesenchymal Stem Cells

Mesenchymal stem cells (MSCs) are versatile cells that possess the ability to self-renew. They can be found in various sources such as human umbilical cord, bone marrow, and adipose tissue. Recent studies have revealed that exosomes released by MSCs exhibit similar characteristics to their parent cells, including the ability to modulate cell migration and proliferation, regulate the immune response, promote tissue regeneration, and exhibit anti-inflammatory effects [48–51].

In obese mice, adipose-derived stem cell- (ADSC-) derived exosomes were found to improve metabolic homeostasis by promoting M2 macrophage polarization, reducing inflammation, and inducing beiging in white adipose tissue (WAT). This was achieved through the transfer of exosomes to macrophages, which induced anti-inflammatory M2 phenotypes by activating arginase-1 through STAT3 carried by the exosomes. M2 macrophages induced by ADSC-derived exosomes also promoted ADSC proliferation and lactate production, favoring WAT beiging and homeostasis in response to high-fat challenge [52]. Collectively, exosomes derived from stem cells have the potential to enhance insulin sensitivity by facilitating insulin signaling and reducing inflammation associated with adipose tissue (Figure 2).

## 7. Clinical Applications of Exosomes in T2DM Management

Exosomes contain various bioactive molecules, such as microRNAs, proteins, and lipids, that can regulate cellular functions. Exosomes have emerged as promising tools for diagnosing and treating various diseases due to their ability to transfer bioactive molecules between cells. The potential clinical applications of exosomes in T2DM management include biomarkers for disease diagnosis and monitoring, development of exosome-based therapies, and exosomes as drug delivery vehicles.

### 7.1. Diagnosis Tools

Exosomes can be easily obtained from bodily fluids like plasma, saliva, breast milk, sweat, tears, and urine. The ability of exosomes to protect cargo from degradation and their dependence on the condition of the human body makes them a potentially valuable diagnostic tool for T2DM. They are potential biomarkers for T2DM diagnosis and monitoring of disease progression.

Notably, phosphoenolpyruvate carboxykinase in urine exosomes indicates the kidney's involvement in gluconeogenesis. This noninvasive marker can aid in identifying early insulin resistance and impaired gluconeogenesis in humans [53]. A clinical study found that specific miRNAs in extracellular vesicles found in human plasma could be used as biomarkers to identify insulin resistance phenotypes in obese individuals. The study discovered four miRNAs (let-7b, miR-144-5p, miR-34a, and miR-532-5p) with a substantial predictive value for insulin resistance [54]. Several other exosomal miRNAs have been found to increase in the plasma or urine of diabetic patients, making them promising biomarkers associated with type 2 diabetes. Examples of such miRNAs include miR-21-5p, miR-375-3p, miR-133b, miR-342, and miR-30, which are all elevated in the serum of diabetic individuals, as well as miR-451-5p, let-7c-5p, miR-362-3p, miR-877-3p, miR-150-5p, and miR-15a-5p, which are increased in the urine of diabetic individuals [55–57]. Also, the levels of miR-7 were considerably higher in individuals with T2DM and T2DM-associated microvascular complications (T2DMC), and most of it was present in the form of exosome-free [58]. The combination of exosomal DLX6-AS1 and PRINS has good diagnostic value for diabetic retinopathy (DR), particularly in males. Exosomal PRINS expression may be a predictive and diagnostic biomarker for DR in females [59].

### 7.2. Exosome-Based Therapeutics

Pretreating mesenchymal stem cell-derived exosomes with melatonin (MT) has enhanced diabetic wound healing by reducing inflammation. This is achieved by activating the PTEN/AKT signaling pathway, increasing the M2 to M1 polarization ratio. MT pretreatment represents a promising approach for treating diabetic wounds [60]. It appears that human umbilical cord mesenchymal stromal cells can alleviate muscle atrophy caused by diabetes and obesity. This is achieved through the enhancement of autophagy mediated by the AMPK/ULK1 signaling pathway via exosomes [61]. Regenerating pancreatic *β*-cells is a potential way to treat some forms of type 2 diabetes, but it does not happen naturally very often. However, bone marrow transplantation (BMT) has been shown to encourage *β*-cell regeneration in an unknown way(s). Additionally, mice with insulin-deficient diabetes were given miR-106b/222 through IV, which increased *β*-cell growth and improved high blood sugar. Therefore, these miRNAs could lead to new treatment options for diabetes [62]. The exosomal microRNA miR-146a, derived from MSCs, can combat inflammation in damaged astrocytes and prevent cognitive impairment caused by diabetes [63]. Nonetheless, M2-exosomes can modulate the osteoimmune microenvironment by reducing the proportion of M1 macrophages and inducing the conversion of M1 macrophages into M2 macrophages via the PI3K/AKT pathway by accelerating diabetic fracture healing [64].

Chronic nonhealing diabetic wounds present a significant clinical challenge, and engineered MSC-derived exosomes can potentially promote wound healing. This study focused on eNOS-rich umbilical cord MSC exosomes (UCMSC-exo/eNOS) modified by genetic engineering and optogenetic techniques. The study found that UCMSC-exo/eNOS significantly improved the biological functions of cells after high-glucose treatment and reduced the expression of inflammatory factors and apoptosis induced by oxidative stress. In vivo, UCMSC-exo/eNOS significantly improved the rate of wound closure and enhanced vascular neogenesis and matrix remodeling in diabetic mice. UCMSC-exo/eNOS also improved the inflammatory profile at the wound site and modulated the associated immune microenvironment, thus significantly promoting tissue repair. This study provides a novel therapeutic strategy based on engineered stem cell-derived exosomes to promote angiogenesis and tissue repair in chronic diabetic wounds [65]. MSC therapy is an innovative approach in diabetes due to its capacity to modulate the tissue microenvironment and regenerate glucose-responsive insulin-producing cells.

After discovering that exosomes can induce biological effects on target cells, a competition to utilize their potential as therapeutic agents commenced. Exosomes can transfer bioactive molecules between cells in vitro and in vivo, providing a promising avenue for developing of novel therapeutic applications. The potential of utilizing exosomes as therapeutic agents is highly promising due to their several advantages. Exosomes can be easily obtained from human cell lines or blood. It can carry siRNA, biomaterials, and drugs to the specific organ for treatment after engineering due to immunological compatibility. Moreover, exosomes can cross the intact blood-brain barrier and are distributed throughout the body, which is a promising vehicle for delivering agents to the brain for metabolism-related neurological disorders.

Overall, we highlight the crosstalk of exosomes and immune cells in the pathogenesis of T2DM. Exosomes derived from immune cells have been shown to possess immunomodulatory properties and can suppress or enhance immune responses depending on their cellular origin and cargo. Exosomes from nonimmune cells, such as adipose tissue and pancreatic *β*-cells, have also been shown to modulate immune responses in T2DM. The potential clinical applications of exosomes in T2DM management include biomarkers for disease diagnosis and monitoring, the development of exosome-based therapies, and exosomes as drug delivery vehicles. It suggests that exosomes represent a promising area of research for developing novel diagnostic and therapeutic strategies for T2DM.

## 8. Challenges and Limitations of the Current Exosomes' Studies

The utilization of exosomes as therapeutic agents and vectors has shown significant promise. However, several challenges and limitations still need to be addressed. These include issues with scalability, standardization of isolation and purification methods, and the potential for off-target effects [66, 67]. The need for more understanding of the mechanisms underlying exosome-mediated intercellular communication hinders their clinical translation. Despite these challenges, continued research and development in this field hold great potential for advancing novel therapeutic applications.

## 9. Conclusions

T2DM is traditionally considered a simple metabolic disorder. However, it is highly associated with immune dysfunction. Exosomes mediate the biocommunications between immune cells and nonimmune cells, which enhance the release of inflammatory factors to accelerate the progression of T2DM and its complications. Meanwhile, exosomes have emerged as a promising therapeutic approach for T2DM due to their ability to transfer bioactive molecules and regulate glucose metabolism. Several challenges and limitations still need to be addressed before exosomes can be used in clinical practice. Future studies should focus on establishing consensus methodologies for the isolation, characterization, and manipulation of exosomes and identifying the mechanisms underlying cargo sorting into exosomes to optimize their therapeutic potential. Additionally, more research is needed to determine the potential off-target effects of exosomes and to improve the scalability and standardization of isolation and purification methods. Despite these challenges, continued research and development in this field hold great potential for advancing of novel therapeutic applications for T2DM.

## Figures and Tables

**Table 1:** Current studies of exosomes from immune cells on immune responses in T2DM.

Source	Contents	Functions	Expression in diabetic status	Immune responses	Experimental model	References
M1-polarized macrophages	miR-212-5p	Targeting SIRT2 and inhibiting Akt/GSK-3*β*/*β*-catenin pathway	Upregulate	Proinflammatory	HFD mice	Qian et al. [33]
High glucose-induced macrophage RAW 264.7 cells	miR-210	Impairing glucose uptake and mitochondrial CIV complex activity and suppressed NADH dehydrogenase ubiquinone 1 alpha subcomplex 4 (NDUFA4) expression in 3T3-L1 adipocytes	Upregulate	Proinflammatory	Obese diabetic mice	Tian et al. [34]
Diabetic bone marrow-derived macrophage	miR-144-5p	Transferring into bone mesenchymal stem cells to regulate bone regeneration by targeting Smad1	Upregulate	Proinflammatory	T2DM mice	Zhang et al. [35]
M2 macrophage	M2-exosomes	Promoting macrophage transformation from M1 to M2 by targeting the PI3K/AKT pathway	Upregulate	Anti-inflammatory	T2DM mice	Wang et al. [64]
M2-polarized macrophage	miR-690	Nadk as a target gene of miR-690	Upregulate	Anti-inflammatory	Obese mice	Ying et al. [36]
Natural killer cell	miR-1249-3p	Targeting SKOR1 to regulate the formation of ternary complex SMAD6/MYD88/SMURF1, which mediates glucose homeostasis by suppressing the TLR4/NF-*κ*B signaling pathway	Upregulate	Anti-inflammatory	T2DM mice	Wang et al. [37]

The exosomes miR-212-5p, miR-210, and miR-144-5p are derived from immune cells and have a role in promoting inflammation in T2DM, whereas M2-exosomes, miR-690, and miR-1249-3p show anti-inflammatory effect.

**Table 2:** Current studies of exosomes from nonimmune cells on immune responses in T2DM.

Source	Contents	Functions	Expression in diabetic status	Immune responses	Experimental model	References
Pancreatic *β*-cells	miRNA-29	Promoting the recruitment and activation of circulating monocytes and macrophages and, hence, inflammation, via miR-29 exosomes in a TRAF3-dependent manner	Upregulate	Proinflammatory	HFD mice	Sun et al. [43]
Adipose tissue	Sonic hedgehog (Shh)	Inducing proinflammatory or M1 polarization of bone marrow-derived macrophages (BMDM) and RAW 264.7 macrophages	Upregulate	Proinflammatory	3T3-L1 adipocytes	Song et al. [46]
Adipose tissue	Adipose tissue exosome-like vesicles	Targeting the macrophages to the liver and adipose tissues, secrete TNF-*α* and IL-6 via the TLR4/TRIF pathway	Upregulate	Proinflammatory	HFD mice	Deng et al. [47]
Adipose tissue	MicroRNA-34a	Targeting the transcription factor KLF4	Upregulate	Proinflammatory	HFD mice	Pan et al. [10]
Adipose-derived stem cells (ADSCs)	ADSC-derived exosomes	Inducing high levels of M2-related Arg-1 and IL-10 and inhibiting macrophage inflammatory responses stimulated by LPS plus IFN-*γ*	Upregulate	Anti-inflammatory	HFD mice	Zhao et al. [52]

The exosomes miRNA-29, Shh, adipose tissue ELVs, and microRNA-34a are derived from nonimmune cells and play a role in promoting inflammation in T2DM, whereas ADSC-derived exosomes show anti-inflammatory effect.
